# Does food marketing need to make us fat? A review and solutions

**DOI:** 10.1111/j.1753-4887.2012.00518.x

**Published:** 2012-10-04

**Authors:** Pierre Chandon, Brian Wansink

**Affiliations:** INSEAD, Fontainebleau, and a member of ICANParis, France; Charles H. Dyson School of Applied Economics and Management at Cornell UniversityIthaca, NY, USA

**Keywords:** consumer behavior, diet, food packaging, health, marketing, mindless eating, obesity, public policy, slim by design

## Abstract

Food marketing is often singled out as the leading cause of the obesity epidemic. The present review examines current food marketing practices to determine how exactly they may be influencing food intake, and how food marketers could meet their business objectives while helping people eat healthier. Particular attention is paid to the insights provided by recently published studies in the areas of marketing and consumer research, and those insights are integrated with findings from studies in nutrition and related disciplines. The review begins with an examination of the multiple ways in which 1) food pricing strategies and 2) marketing communication (including branding and food claims) bias food consumption. It then describes the effects of newer and less conspicuous marketing actions, focusing on 3) packaging (including the effects of package design and package-based claims) and 4) the eating environment (including the availability, salience, and convenience of food). Throughout, this review underscores the promising opportunities that food manufacturers and retailers have to make profitable “win-win” adjustments to help consumers eat better.

## INTRODUCTION

Biology and natural selection have created strong food preferences. Individuals around the world want easy access to a variety of tasty, convenient, inexpensive, and safe foods that can be eaten in large quantities. By catering to, and stimulating, these biological interests, food marketers have been accused of contributing to the growing problem of global obesity.^1–5^ After all, the food industry (which includes food and beverage producers, as well as retailers, restaurants, and food services companies) employs savvy and creative marketers who have pioneered many of the tools of modern marketing.^6,7^ At the same time, it is important to understand that the marketers and the executives who guide them are torn between satisfying the desires of various consumers, the demands of their shareholders, and the concerns of public health organizations, which largely perceive the food industry as the new tobacco industry (because both industries have used similar tactics, such as emphasizing personal responsibility, massive lobbying, pre-emptive self-regulation, etc.).^8,9^ For these reasons, it is useful to review and integrate much of the overlooked evidence on how food marketing influences food intake and to examine how food marketers could continue to grow their profits without growing their customer's body mass index (BMI).

This review article examines and integrates the literature from marketing, consumer research, and related social science disciplines, which is not in the commonly referenced databases for health and medicine, such as PubMed, and is therefore often unknown to nutrition researchers. By incorporating this information, this review updates the existing reviews in the field,^10,11^ which are rapidly becoming outdated given the breadth of more current research. For the purpose of this review, marketing is defined in accordance with the definition of the American Marketing Association as “the activity, set of institutions, and processes for creating, communicating, delivering, and exchanging offerings that have value for customers, clients, partners, and society at large.” This article focuses on the direct effects of marketing activity under the direct control of food marketers, often referred to as the 4 Ps of “product,”“price,”“promotion,” and “place.” Specific focus is placed on the factors that influence how much consumers eat, and in particular, whether they overeat (which is defined as eating more than one realizes). Yet, it is important to remember that food/energy intake is not synonymous with weight gain, let alone obesity.^12^ Because of this review's focus on marketing and food intake, many influencers of food intake that are not under the direct control of food marketers are excluded (e.g., physical activity, pro-social marketing, personal, cultural, and social norms about food, eating, dieting, incidental emotions, etc.).

Food marketers influence the volume of food consumption through four basic mechanisms that vary in their conspicuousness. 1) The short- and long-term price of food, as well as the type of pricing (e.g., a straight price cut or quantity discount), can influence how much people purchase and eventually consume. Pricing efforts are generally conspicuous and lead to deliberate decisions. 2) Marketing communications, including advertising, promotion, branding, nutrition, and health claims, can influence a consumer's expectations of the sensory and non-sensory benefits of the food. Marketing communications comprise the most recognized form of influence and the one most closely scrutinized by marketing and non-marketing researchers. The influence of marketing communication can sometimes be as conspicuous as price changes, but consumers are not always aware of some of the newest forms of marketing communication (e.g., “advergaming,” package design, or social media activities) and, even when they are aware of the persuasive intent behind these tools, they may not realize that their consumption decisions are being influenced. 3) The product itself, including its quality (composition, sensory properties, calorie density, and variety) and quantity (packaging and serving sizes) also influence in a variety of ways how much of the product consumers eat. This area has been frequently researched as marketing communication. 4) The eating environment, including the availability, salience, and convenience of food, can be altered by marketers. Compared to the breadth of the domain, this is the least frequently studied area, yet it is the one most likely to be driven by automatic, visceral effects outside the awareness and volitional control of consumers.

## PRICING: HOW LONG- AND SHORT-TERM PRICE REDUCTIONS STIMULATE INTAKE

Some food products like milk, meats, fruits, and vegetables are often sold as commodities. With commodities, short-term prices are determined by supply and demand on world markets and long-term price changes are determined by efficiency gains in the production, transformation, and distribution of food rather than by marketers. The most notable change in this respect is the relative steep decline in the price of food over the last 50 years, particularly for branded, processed foods that are high in sugar and fat, and for ready-to-eat foods, which are prepared away from home.^13–17^

Yet most food products are *not* commodities; instead, they are branded products that are differentiated in the eyes of consumers thanks to the ways in which they are advertised, formulated, packaged, distributed, and so on. With these branded products, marketers can establish their own price depending on which consumer segment they wish to target. Advances in marketing segmentation have enabled companies to direct price cuts to only the most susceptible consumer segments, which increases their efficiency. Table 1 summarizes key findings about the effects of price on overeating, innovative solutions tested by marketers to mitigate its effects, and suggestions for using price to improve food consumption decisions.

**Table 1:** Pricing and consumer welfare.

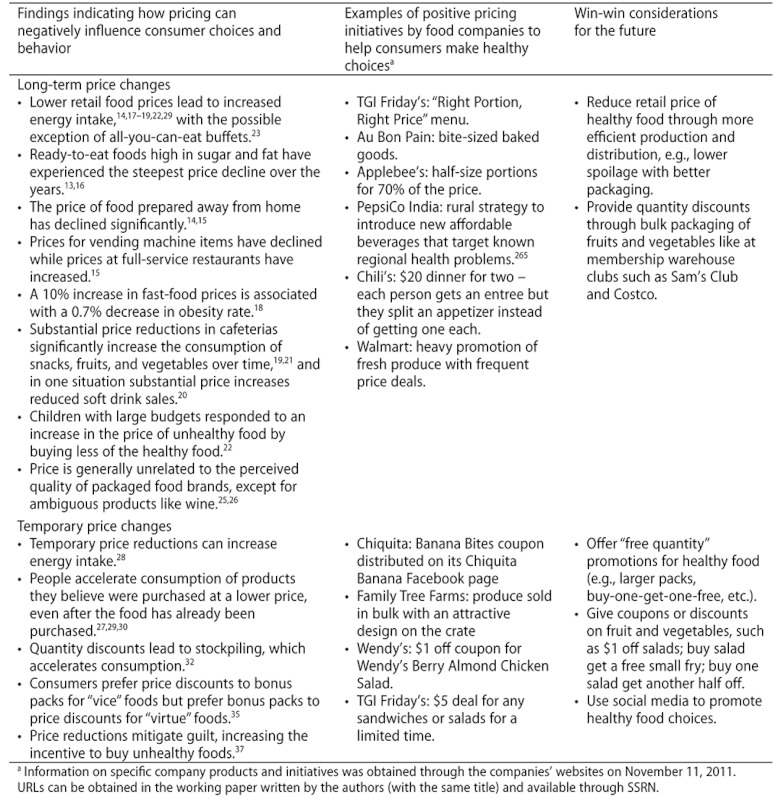

### Effects of long-term price changes

Econometric studies suggest that lower food prices have led to increased energy intake.^13–17^ Even though the average price elasticity of food consumption is low (−0.78), it can be quite high in some categories (e.g., −1.15 for soft drinks) and for food prepared away from home. For example, one econometric study^18^ using data from the 1984–1999 national Behavioral Risk Factor Surveillance System found that a 10% increase in prices at fast-food and full-service restaurants was associated with a 0.7% decrease in the obesity rate.

These conclusions are reinforced by the results of randomized controlled trials which demonstrate the causal effects of price changes. Longitudinal field experiments in cafeterias^19–21^ have found that price changes above 25% significantly influence consumption of beverages or snacks, but also of fruit and vegetables, and they have stronger effects than nutrition labeling, which sometimes backfire because of negative taste inferences. One of the most thorough studies^22^ also varied food budgets over time and found strong and comparable same-price elasticity in two studies for healthy (−1 and −1.7, respectively) and unhealthy (−0.9 and −2.1, respectively) foods. In contrast, the cross-price elasticities were four times smaller and only occurred when children had a very low budget, showing that children do not consider healthy foods to be a substitute for unhealthier ones.

The only exception to the rule that higher prices reduce consumption comes from a study showing that higher prices at an all-you-can-eat pizza restaurant led to higher consumption of pizza, probably because of the psychology of “sunk costs,” which leads people to try to eat “their money's worth.”^23^ Interestingly, monetary (and normative) rewards do not seem to have any adverse effects on children's intrinsic motivation for the food.^24^ In general, consumers appear to have learned that lower-priced foods are as hedonically satisfying as higher-priced foods, with the exception of a few categories, such as wine, for which determining good taste is ambiguous.^25^ For example, in a recent study, Austrian consumers thought that price was unrelated to the quality of foods, which is not surprising given that the correlation between price and quality in that country was estimated by experts at only 0.07.^26^

### Effects of temporary price promotions and quantity discounts

Until recently, it was believed that price promotions simply shifted sales across brands or across time. However, it has now become clear that temporary sales promotions can lead to a significant increase in consumption.^27,28^ Probably the best evidence of this comes from a randomized controlled field experiment involving 1,104 shoppers.^29^ This study found that a 12.5% temporary price discount on healthier foods increased the purchase volume of these foods by 11% among the low-income consumers who received the coupons. The effect persisted even 6 months after the promotion had been stopped. In comparison, nutrition education and suggestions for substituting healthier food for less healthy food had no effect, whether alone or combined with the price discounts. However, the discounts on healthy food did not reduce purchases of unhealthy food.

Price deals can influence the speed of consumption even when the food has already been purchased (such as by another family member) and is, therefore, an irreversible sunk cost; this should not, in theory, influence consumption because the cost cannot be recovered, no matter when, or how quickly, the food is consumed. Nevertheless, studies have found that people accelerate the consumption of products perceived to have been purchased at a lower price.^30^ This happens because a reduced past price is seen as an indication that the product will be discounted again in the future^31^ or simply because the reduced sunk cost means that consumers feel they do not have to wait for a special occasion to consume the product perceived to be cheaper.^32^

Marketers also reduce the relative price of food by offering quantity discounts with larger package sizes or multi-unit packs, which is a powerful driver of supersizing.^33^ Although there are exceptions, most studies found that quantity discounts generally lead to stockpiling and increased consumption, especially for overweight consumers.^27,34^ One study found that during weeks in which multi-unit packages were purchased, consumption of orange juice increased by 100% and cookies by 92%, but there was no change in consumption of non-edible products.^32^ The authors replicated this effect in a field experiment in which the quantity of food was randomly manipulated while keeping its price constant; they found that large purchase quantities influenced consumption by making the food salient in the pantry or fridge, and not just by reducing its price.

Beyond the degree of the incentive, the form of the promotion and the payment mechanism can also influence energy intake. One study suggests that consumers prefer price discounts to bonus packs for guilt-inducing “vice” foods, but preferred bonus packs to price discounts for “virtue” foods because it is easy to justify buying them in larger quantity.^35^ By definition, “vices” are foods that are preferred when considering only the immediate consequences of consumption and holding delayed consequences fixed, whereas the opposite is true for “virtues.”^36^ The greater difficulty of justifying purchases of unhealthy foods also explains why they are more likely to be purchased when people pay for their grocery purchases via credit card than when they pay cash – a more painful form of payment which elicits a higher need for justification.^37^ On the other hand, people are more likely to purchase and consume indulgent high-calorie ice-creams when paying cash than when paying with a credit card,^38^ possibly because in this case they have the opposite goal of rewarding themselves.

### Summary

Overall, all the studies reviewed here clearly show that pricing is one of the strongest – if not *the* strongest – marketing factors predicting increased energy intake and obesity, and this is why lower-income consumers are predominantly affected by these conditions. Conversely, the power of pricing means that it holds the key to many of the “win-win” solutions detailed in Table 1. However, price is not the only determinant of food choices and it cannot alone explain rising obesity rates.^18^ Unlike price, which arguably influences consumption through deliberate processes that people are aware of, food communication influences food perceptions and preferences often beyond volitional control and sometimes outside conscious awareness.

## PROMOTION: HOW MARKETING COMMUNICATION STIMULATES INTAKE

Advertising and promotions are one of the most visible and studied actions of food marketers. They include advertising, both on traditional media channels and on non-traditional non-media channels, such as online, in-store, in movies, television programs or games, sponsorship or organization of events, in the street, and so on. Food marketers also communicate in more indirect ways by branding the entire product category (e.g., the “Got Milk?” campaign), the ingredients (e.g., acai), and by making nutrition or health claims in their advertising or on their packages. These claims are distinct from the mandatory nutrition information about calories, nutrient levels, and serving sizes, whose effects are reviewed elsewhere.^39–44^Table 2 summarizes the effects of marketing communication and shows how they can also improve food choices.

**Table 2:** Marketing communications (promotion) and consumer welfare.

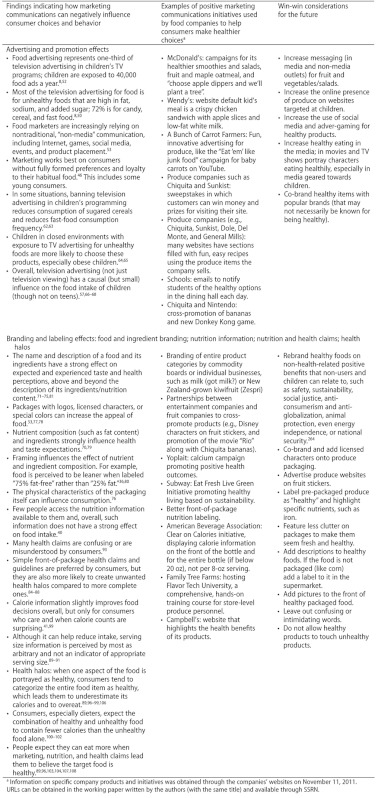

Marketing communication informs people about product attributes, like the price or where it can be purchased. Marketing communication also increases awareness of the brand and food, which leads consumers, particularly children, to try fewer foods and to only search for brands they already know rather than the brand that would have the highest nutritional and hedonic qualities.^45–47^ Moving beyond awareness, communication enhances a consumer's expectations of the sensory and non-sensory benefits (such as the social and symbolic value) associated with the purchase and consumption of a particular food. Even if it fails at changing the expected benefits of consumption, marketing communication can influence the importance of these benefits, for example, by making taste a more important goal than health. This may explain why nutrition ranks last in surveys of the drivers of food choices, after taste, cost, and convenience.^48,49^

### Advertising and promotion effects

The food industry is among the top advertisers in the US media market. Children and adolescents are exposed to increasing levels of television advertising, mostly for nutritionally poor snacks, cereals, candies, and other food with a high fat, sodium, or added sugar content.^50–52^ As with all consumer goods marketers, food marketers are diverting budgets from television, print, radio, or outdoor advertising to more recent forms of communication on new media (including web sites, all types of video games, social networks, product placement, point-of-purchase advertising, etc.) and through packaging, direct marketing, public relations, and event sponsorship.^53^ The message communicated in these ads is that eating these foods is normal, fun, and socially rewarding.

Given how much food marketers spend on communication, and particularly television advertising, it is surprising that a link between television advertising and energy intake is still perceived to be controversial by some. Some researchers contend that television advertising only affects brand preferences and not overall energy intake, while others demand an extremely high bar before any conclusion can be drawn.^54–56^ Part of the explanation for the duration of the controversy is that, unlike other factors, such as price or portion size changes, advertising is a complex multi-dimensional intervention. Two campaigns can vary in their reach, frequency, scheduling, targeting, message strategy, and execution. In combination, this makes it difficult to conclusively estimate reliable effects using non-experimental real-world data.

*Television viewing or television advertising?* The correlation between television viewing and obesity is well established. Television viewing is associated with unhealthy snacking. Eating in front of the television also distracts, and therefore slows awareness of satiety.^57–59^ Although television viewing also reduces calorie expenditures directly (by displacing physical activity) or indirectly (by advertising cars, games, and indoor toys that promote a sedentary lifestyle), studies suggest that the effects of television viewing on calorie expenditure are too weak to materially impact obesity.^57,60,61^ Still, these studies cannot disentangle the effects of television *viewing* from the effects of television *advertising*.

One of the reasons it is difficult to estimate how television advertising influences energy intake is because there is very little natural variation in real-world exposure to television advertising for food, requiring one to make many statistical assumptions. In this context, probably the most convincing studies use real-world data from Québec's ban on television advertising aimed at children in French-speaking television networks. A first study^62^ showed that the ban reduced the quantity of children's cereals in the homes of French-speaking children in Québec, but not for English-speaking children who continued to be exposed to the same amount of food advertising through US television stations. Another study^63^ concluded that the Québec ban also significantly reduced fast-food consumption because French-speaking families in Québec with children eat less often in fast-food restaurants than English-speaking families with children, but no such difference are found between families without children or between French- and English-speaking families living in Ontario. These results are corroborated by other experimental studies in schools and summer camps, which showed that exposure to television advertising for unhealthy foods increased the likelihood that these foods would be chosen on a single consumption occasion as well as for longer time periods, and that the largest effects occurred among obese children.^64,65^

In summary, reviews of this literature suggest that food advertising moderately influences the diet of children (though not of teens). There is not, however, enough evidence to rule out alternative explanations regarding its effects on obesity itself.^57,66–68^ It is also suggested that food advertising interacts with other marketing factors, such as price promotions, and with factors not directly under the control of marketers, such as social norms, to influence obesity to a degree which would be very hard to establish precisely.

### Branding and labeling effects

**Food and ingredient branding.** Branding is the creation of names, symbols, characters, and slogans that help identify a product and create unique positive associations which differentiate it from the competition and create additional value in the consumer's mind.^69^ The name of the food (brand name or generic category name) has a strong influence on how consumers' expectations of how tasty, filling, or fattening the food will be, which are often uncorrelated with reality.^70,71^ Well-known brands, but also simple descriptions like “succulent,” can influence taste expectations, consumption experience, and retrospective evaluations of the taste, and then lead to increased sales, especially for non-experts.^72–74^ For example, a recent study^75^ showed that branding the same food as a “salad special” versus “pasta special,” or as “fruit chews” versus “candy chews” increased dieters' perceptions of the healthfulness or tastiness of the food as well as its actual consumption. Interestingly, name changes had no impact on non-dieters and disappeared when dieters were asked to consider the actual ingredients (versus the name), and when looking only at dieters with a high need for cognition. Consumers also form expectations about the product from any attribute associated with the product, from the presence of licensed or brand-owned character,^53^ to the firmness of its container.^76^

Beyond the name of the food, communication about the nutrient composition and the presence (and number) of specific macro nutrients or ingredients (especially fat content, but also energy density, fiber, sugar content, unfamiliar long-worded ingredients, and so on) can strongly impact food expectations.^77–79^ As with any communication, the framing of the information matters also for nutrition information. Food is perceived to be leaner and higher quality when labeled “75% fat-free” than “25% fat.”^36,80^ For example, vinegar improves the taste of beer, but only when it is described as a “special ingredient,” not when it is described as vinegar, and only when the description is provided prior to the consumption.^81^ This suggests that branding influences the interpretation of the sensory experience and does not just modify the retrospective interpretation of the experience. In fact, marketing descriptions of a milkshake as “indulgent” or “sensible” influences physiological satiation, as measured by gut peptide ghrelin.^82^ Neuroimaging studies confirm that these marketing actions influence not just self-reported liking, but also its neural representations, suggesting that these effects are not merely influenced by social cues and that marketing actions modify how much people actually enjoy consuming the food.^25^

**Health and nutrition claims.** Although nutrition and health claims are regulated, the decision of whether or not to use them rests with the food marketers. In past years, marketers have become increasingly likely to make heavy use of nutrition claims (including “low fat” or “rich in omega 3”), “structure-function” claims (“proteins are essential for growth”), health claims (“supports immunity”), vague unregulated claims or health sales (including “smart choice” or “good for you”), or the use of third-party ratings or endorsements (including “Kosher,”“Halal,”“organic,” or the heart check mark of the American Heart Association). Some of these claims can improve brand evaluation and sales, although these effects are not universal and are influenced by comparisons with other foods in the same category and by how they influence taste expectations.^43,83^

Studies have shown that simpler, more prescriptive health claims, such as color-coded traffic lights, have stronger effects.^84,85^ A field experiment found that simple color coding of cafeteria foods with a green, yellow, or red label (for “healthy,”“less healthy,” and “unhealthy” foods) improved sales of healthy items and reduced sales of unhealthy items.^86^ Providing category benchmarks for each ingredient and nutrient (average or range) helps consumers process nutrition information, while summarizing information in a graphic format is particularly helpful for illiterate consumers.^87,88^ Food marketers could also choose to provide information about recommended serving sizes (which is only mandatory in the United States). One study found that, although adding serving size information reduced granola intake for both overweight and normal-weight consumers, it had no impact if the granola was labeled as “low fat.”^89^ The same authors found that promoting smaller serving sizes did not influence intake or satiety ratings, especially among overweight people. This could be because most consumers think that the entire content of the package is the appropriate serving size and perceive USDA serving sizes as an arbitrary unit designed to allow a comparison of nutrition facts across products, rather than as a general guide to how much people should consume.^90,91^

Beyond evaluating whether health claims are scientifically true, an important question to examine is how they are understood by consumers. Recent reviews have identified many sources of confusion.^92–94^ First, although the relationship between any nutrient and health is almost always curvilinear, consumers expect it to be monotonic (“more is better”). Second, consumers may not realize that they are already taking too much of a particular nutrient (e.g., protein intake in Western countries). Third, wording can be misleading; such as when “provides energy” is understood as “energizing.” Finally, some claims are based on flimsy science, or they overstate research findings. For these reasons, health claims are likely to become even more regulated, and to be only allowed for general products as opposed to specific brands, for example.

**Health halos.** The branding and labeling of food often operate by relying on people's natural tendency to categorize food as intrinsically good or bad, healthy or unhealthy, regardless of how much is eaten.^95^ When branding and labeling efforts emphasize one aspect of the food as healthy, it can lead to a “health halo,” whereby people generalize that the food scores highly on all nutrition aspects, including weight gain.^96–98^ In one study,^89^ we found lower calorie estimations for granola than for M&Ms, a product with the same calorie density but considered less healthy than granola. The same study also found that labeling both products as “low fat” reduced calorie estimation and increased the amount that people served themselves or consumed, especially for people with a high body mass index. In another study,^96^ we found evidence for health halos created by the name of a restaurant or the food available on a restaurant menu. For example, meals from the sandwich chain SUBWAY® were perceived to contain 21% fewer calories than same-calorie meals from McDonald's. These results were replicated with other foods and restaurant brands.^99^

Related studies showed that adding a healthy food to an unhealthy food could lead to calorie estimations that were lower than for the unhealthy food alone. For example, one study found that a hamburger alone was perceived to have 761 calories but the same hamburger and a salad was thought to have only 583 calories.^100^ This “negative calorie” illusion created by adding a healthy food to an unhealthy food is particularly strong among people who are on a diet.^101^ Different biases, or contrast effects, occur when people estimate calories sequentially instead of simultaneously.^102^

Overall, the finding that people expect that they can eat more, and do, when marketing actions lead the food to be categorized as healthy is robust and is replicated independently of people's BMI, gender, or restrained eating.^103,104^ This boomerang effect seems to occur because people feel that they can eat more of the healthy food, or can eat more unhealthy, but tasty, food after choosing healthy food without guilt and without gaining weight.^96,105,106^ In fact, simply considering the healthier option without actually consuming it, or forced choice of healthy food can be enough to allow some consumers to vicariously fulfill their nutrition goals, which makes them hungrier and entices them to choose the most indulgent food available.^107,108^

To fully understand the effects of health claims, however, we must look at their impact on choice and purchase and not just on consumption volume when they are freely provided. When examining purchases, the results are mixed. First, studies have shown that people generally expect food presented as “unhealthy” to taste better, and that these effects persist even after actual intake,^109^ although another study found this only among people who are not on a diet.^75^ These results, coupled with the earlier findings that taste expectations are the strongest driver of food choices, imply that positioning food as healthy may not necessarily increase total energy consumption if the higher intake per consumption occasion is compensated by fewer consumption occasions (or fewer consumers).

The net effect of health claims probably depends on brand and individual characteristics, and is stronger for some claims than others. For example, differences in taste expectations about food, specifically when described as “low fat,” as opposed to branded as “healthy” in general, have been found between men and women,^110^ and mostly influence unfamiliar brands. It is also unlikely to influence foods strongly categorized as healthy or unhealthy. This could explain the null effect of some of the studies and some of the earlier opposite findings.^111,112^ The negative association between health and taste is less pronounced in Europe, where people tend to associate “healthy” with freshness and higher quality, and thus sometimes healthier can be tastier.^113,114^

## PRODUCT: HOW MARKETING STIMULATES INTAKE BY CHANGING THE FOOD ITSELF

Although marketing is most readily associated with communication and pricing, marketers are also closely involved with product development decisions. This includes making decisions about the “quality” of the food and also its “quantity.” The effects of changes in the product on overeating are summarized in Table 3. This table also shows how some food marketers have found ways to mitigate these changes and provide avenues for further win-win strategies.

**Table 3:** Product and consumer welfare.

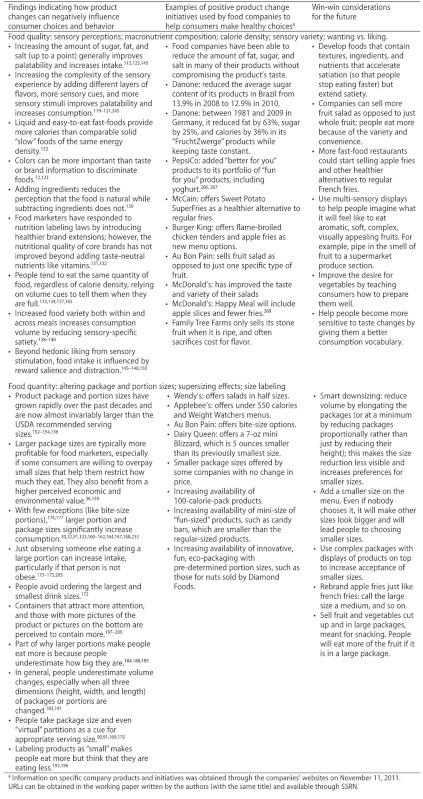

### Product quality: effects of the composition, sensory, and nutritional properties of the food

In addition to being a source of nourishment, food is a source of hedonic pleasure and stimulation. Hence, it is not surprising that one of the primary goals of food marketing is to improve the palatability of the food. At a basic level, palatability generally increases energy intake because people in rich countries can choose to eat only what they like.^115^ Although improving palatability and the sensory and nutritional properties of food are largely driven by advances in food science, marketing plays an important role because it helps incorporate the expressed and latent desires of consumers and, above all, the role of perception. For example, advances in market research can correct for the fact that some people may not like a given amount of sweetness simply because they are not as sensitive to it as much as others or because they have a different interpretation of a scale label such as “extremely sweet.”^116,117^ This is particularly important because taste perception and preferences are not the same for people with a high and low BMI.^118^

**Food composition.** Flavor is a seamless combination of taste and predominately smell, but it is also enhanced by adding different layers of flavors; combining different forms (solid or liquid), textures, colors, or temperatures also influences flavor perceptions due to multisensory taste integration as well as consumers' expectations.^119–121^ These factors can directly impact energy intake independent of their impact on flavor. People tend to consume more calories from liquid than from comparable solid foods of the same energy density because the lower bite effort and shorter sensory exposure postpone satiation.^122^

Because people associate certain colors with certain foods and flavors, food marketers have long used colors to improve taste expectations. For example, some colors, especially those with strong flavor expectations, can influence the perceived sweetness of food and play a very important role in helping consumers discriminate between different foods, sometimes bigger than the role played by taste or brand information.^72,123^ Even advertisements that evoke multiple sensory experiences can enhance taste perceptions.^124^

Up to a certain level, adding sugar, fat, and salt, especially in combination, improves palatability, but does not increase the satiating power of the food in the same proportion.^125,126^ Accordingly, food marketers have expanded the supply of food rich in fat or added sugar, such as sweetened beverages, which have accounted for a large proportion of the added supply of calories in recent decades.^127,128^ Even though it is true that the percentage of calories consumed from fat has declined in the United States, this percentage decrease is the result of an increase in total energy intake; fat consumption itself has not decreased.^129^ Interestingly, adding ingredients reduces the perception that the food is natural, which is an important criteria for food choices, whereas subtracting ingredients (e.g., skim milk) does not.^130^

Food marketers have changed the composition of foods not just to increase palatability but also to respond to public concerns about a particular ingredient or to regulatory changes. Surprisingly perhaps, responses to mandatory nutrition labeling have been mixed. One study suggested that the Nutrition Labeling and Education Act of 1990 led food marketers to improve the level of taste-neutral positive nutrients, such as vitamins, in their core brands (especially those with a weak nutritional profile) and to introduce healthier brand extensions with similar levels of positive nutrients but with lower levels of negative nutrients, especially in junk food categories.^131,132^ However, despite these advances, the average nutritional quality of food products sold in grocery stores had actually worsened compared to pre-NLEA levels and compared to similar food products unregulated by the NLEA.^132^ This is largely driven by established brands, which account for a large portion of people's diet (e.g., dinner food) and whose nutritional quality has slightly deteriorated. This may be because companies are afraid of reducing levels of negative nutrients (e.g., fat or sodium) in their flagship brands for fear that it may decrease flavor expectations and because companies prefer to compete on taste rather than on nutrition, which can now be more easily compared.

**Calorie density and sensory variety.** The biggest share of marketing budgets, and most new product introductions, tend to be for calorie-dense foods with a variety of flavors.^2^ Unfortunately from a public health perspective, it is well established that calorie density – the number of calories per unit of food – increases energy intake over the short term, such as during an afternoon snack. This happens because people prefer calorie-dense food and tend to eat the same volume of food regardless of its calorie density.^133–135^ One of the explanations for this finding is that, instead of paying attention to internal signals of satiation, they focus on external signals, which are often biased.^136^ In one study, unsuspecting diners were served tomato soup in bowls that were refilled from tubing that ran under the table and up into the bottom of the bowls. People with varying BMI levels eating soup from these “bottomless” bowls ate 73% more soup than those eating from normal bowls, but these diners estimated that they ate only 4.8 calories more.^137^

It is well known that food variety, both within and across meals, increases consumption volume because it reduces sensory-specific satiety within a meal and it reduces monotony across meals.^138–140^ The variety effect is independent of macronutrient content and energy density; it is also independent of individual characteristics such as gender, weight, and dietary restraints, and is only somewhat reduced with age. Research in marketing has focused on perceived (versus true) variety. It has shown that increasing the number of colors and the organization, duplication, and symmetry of an assortment can influence perceived variety, which then influences the perceived quantity of food and, ultimately, how much food is chosen.^141–144^ Food marketers have explored many ways to increase perceived variety, including distraction, varying condiments, or giving people illusory choice over what they eat.^138^

**Wanting versus liking.** Despite the links between sensory stimulation, palatability, and consumption, the availability of tasty, highly palatable foods is neither a necessary nor a sufficient cause of over-consumption.^145,146^ While a highly satisfying meal can lead one person to not want to eat dessert, it can trigger the desire in another person. In fact, highly palatable food samples actually enhance subsequent consumption of similar foods and may prompt people to seek any other type of rewarding food.^147^ Even then, people eat beyond the level at which their appetite is satisfied, which is why people eat and drink less when asked to focus on taste satisfaction.^148^ Conversely, mental stimulation can create habituation. Simply imagining eating 30 pieces of cheese reduces consumption, increases satiation for the imagined food, and reduces subsequent wanting for the food, but not its hedonic liking.^149^

More generally, there is converging evidence that food decisions are influenced by motivational “wanting”– the salience or reinforcement value of eating – and not just by hedonic “liking”– the pleasure derived from sensory stimulation.^150,151^ So although there is no doubt that marketing has played a role in developing more complex, palatable, and rewarding foods which people cannot easily resist or stop eating,^2^ the hedonic effects of sensory properties are again just one of many drivers of energy intake.

### Product quantity: altering package and serving sizes

**Trends in serving and package sizes.** With the exception of some specific foods that must be sold in standardized sizes (e.g., wine and liquor), most food and beverage manufacturers are free to choose the size and description (e.g., “medium” or “value” size) of the packages and servings that they sell. Product package and serving sizes have grown rapidly over the past decades and are now almost invariably larger than the USDA recommended serving sizes.^152–154^ While this is a trend in much of the developed world, such “supersizing” is particularly common in the United States and has been identified as one reason why obesity has increased faster in the United States than in other developed countries.^155–157^

Larger package sizes almost always have lower unit prices (by volume or weight), except in the rare instances when there is more competition on the smaller sizes or when smaller sizes are used as loss leaders by retail stores.^158^ Marketers can reduce the unit price of larger products and hence increase consumer value because of their lower packaging costs. More importantly, larger servings and packages provide greater absolute margins because the marginal cost of the extra food is often minimal compared to its perceived value for the consumer. For food retailers and restaurants with high fixed costs (such as high real estate, labor, or marketing costs), reducing serving sizes, and hence average consumer expenditure, would require a huge increase in traffic to break even – which is why the few restaurant chains that have tried this tactic have mostly stopped promoting these items or stopped offering them altogether. In fact, it can even be optimal for food marketers to price the incremental quantity below its marginal cost if their products are bought by two distinct consumer segments: one willing to pay more for smaller portion sizes that help them control their intake, and the other unconcerned about overeating and willing to buy larger quantities to obtain the lower unit price.^36,159^ As a result, larger package sizes are typically more profitable for food marketers, and they benefit from a higher perceived economic and environmental value, a win-win in all aspects but convenience and consumption control.

**Supersizing effects.** There is considerable evidence that, with the exception of children under 3 years of age who still self-regulate naturally, larger package and serving sizes significantly increase consumption.^30,32,91,160–163^ These studies have shown that the increased energy intake due to supersizing (as well as the decrease in energy intake due to downsizing) often reach a 30% change in calorie intake and are not followed by caloric compensation for up to 10 days.^164–166^ Supersized servings can even increase the consumption of bad-tasting foods, such as stale 5- and even 14-day-old popcorn.^167,168^

Even “virtual” serving sizes can influence consumption. Simply adding unobtrusive partitions (e.g., colored papers in between the cookies inside the package or a red Pringle chip between every seven yellow ones in a tube) can reduce intake.^169,170^ However, partitioning may only work when people pay attention to the partition. One study^171^ found that 93% of the purchasers of a king-size pack containing two single-serving candy bars intended to consume both within one day, often because they had not noticed that smaller sizes of candy bars were available for purchase. This is consistent with earlier results indicating that people take package size as a cue for appropriate serving size.^90,91^

The effects of package size on consumption are strongly influenced by the range of the other sizes available and by the serving size chosen by other consumers. One study^172^ found that people in hypothetical choice scenarios avoided the largest or smallest drink sizes. Such aversion to extremes causes consumers to choose larger size drinks when the smallest drink size is dropped or when a larger drink size is added to a set. Social modeling studies have shown that larger package and serving sizes can also have an indirect, passive, impact on energy intake, since people tend to imitate how much other people choose, particularly if the person that they have observed is not obese.^173–175^

There are important exceptions to this rule, however. Small units of products such as 100-calorie packs may increase consumption volume on one consumption occasion more than regular-size packs for hedonic products and when people's self-regulatory concerns have been activated, or for restrained eaters.^176,177^ These studies show that, unlike larger package sizes, small units “fly under the radar” and encourage lapses in self-control because the consumption of these small packages fails to activate healthy eating goals. However, these effects do not seem to hold for long periods, whereupon small sizes do lead to reduced calorie intake.^164,178^

One of the explanations for why large packages and servings increase consumption is the social norm that people should clean their plate.^153,179^ However, this norm cannot explain why large packages also increase the pouring of inedible products such as shampoo, cooking oil, detergent, dog food, and plant food. Nor does it explain why large packages of M&Ms, chips, and spaghetti increase consumption in studies where even the smaller servings were too large to eat in one sitting.^30,163,180^ Another explanation is that larger serving sizes are used as an indication of the “normal” or “appropriate” amount to consume. Even if people do not clean their plate or finish the package, the large size presented to them gives them the liberty to consume past the point where they might otherwise stop with a smaller but still unconstrained supply.^91^ This explanation is consistent with the finding that supersized servings increase energy intake even when people eat in the dark.^181^ Other studies have shown that people associate larger servings with higher status and that people are therefore more likely to supersize when they want to signal status, for example, when they are made to feel powerless.^182^

A final, and important, reason is that people are simply unaware of how large the supersized servings and packages are.^183,184^ Information about food size, volume, or calorie content is not always easily available (such as in restaurants or at home once the food is no longer in its original packaging). Even in retail settings, where size information is available (on the front of the packages or on the shelf tags), few people read it, preferring to rely on visual estimations of the package's weight or volume to infer the amount of product that it contains.^185,186^ Many studies have shown that people's perception of serving sizes is inelastic (it changes more slowly than it should).^187–191^ On average, a 100% increase in serving size only looks like a 50–70% increase. As a result, whereas small servings tend to be accurately estimated, large servings are greatly underestimated.^188^ These perceptual biases are very robust and even trained dieticians exhibit a strong diminishing sensitivity as the size of the meal increases. They are independent of the individual's BMI or interest in nutrition, and they have been replicated by other researchers across a variety of food categories.^99^ Stated simply, meal size, not body size, explains serving size errors. People with a high BMI tend to underestimate their calorie intake more than people with a low BMI^192^ because they tend to select larger meals, not because they are intrinsically worse (or biased) size estimators.^189^

**Size labeling.** The size labels used for food and beverages (such as “short” or “large” and also “biggie” or “petite”) have acquired meanings among consumers, who are generally able to rank order them accurately.^193^ In reality however, these labels mask huge discrepancies because a small size from one restaurant or brand can be larger than a medium size from another.^194^ For example, McDonald's abandoned its supersize 42-oz beverages and 200-g fries, while other fast-food chains retained the serving size but simply renamed the “king” a “large.”^51,195^ These labels are important because they influence size perceptions, preferences, and actual consumption. One study^196^ found that “labeling down” (labeling a large serving “medium”) had a stronger impact on size perception than “labeling up” (labeling a small serving “large”). In addition, these authors found that smaller labels made people eat more but think that they eat less.

A few studies have shown that marketers can influence impressions of size by changing the visual representations on the package itself. Containers that attract more attention are perceived to contain more product.^197^ Two recent studies^198,199^ showed that people expected packages with pictures of the product on the bottom or on the right of the package to be heavier. Finally, simply showing more products on the packaging has been shown to increase size perception and consumption, especially when consumers are paying attention.^200^ It is important to note that most of these studies were conducted in lab settings or in homes and not in in-store environments. Still, the key conclusion is that the quantity of food, and not just its quality, can have large effects on short-term intake and that consumers are largely unaware of these effects.

## PLACE: HOW MARKETING CHANGES TO THE EATING ENVIRONMENT STIMULATE INTAKE

In the same way that food is more than nourishment, eating is more than food intake. It is a social activity, a cultural act, and a form of entertainment. Paradoxically, eating is also mostly a mindless habitual behavior that is strongly influenced by the environment, often without volitional input.^201,202^ In this context, the most subtle and perhaps the most effective way marketing influences consumption is by altering the eating environment and making food accessible, salient, and convenient to consume. As for the other ways food marketing can influence overeating, Table 4 summarizes the key findings as well as existing and new solutions to reverse the effects of marketing changes to the eating environment.

**Table 4:** Eating environment (place) and consumer welfare.

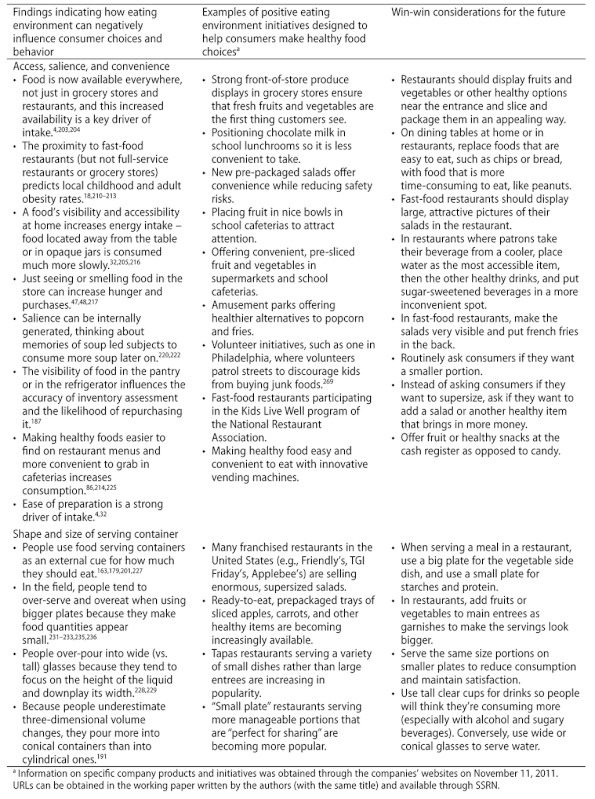

### Access, salience, and convenience

**Access.** One of the biggest goals of food marketers is to facilitate access to food by making food easier to purchase, prepare, and consume. Obviously, food availability is a key factor since food that is not available cannot be consumed.^203^ In addition, the sheer availability of a variety of palatable foods can derail the homeostatic system designed to regulate food intake.^2^ For example, one study found that overweight men on a 3,000 calorie diet did not stick to their diet and consumed an average of 4,500 calories when given access to two free vending machines.^204^ This pattern also holds for healthy foods.^205^

On a more general level, convenient, ready-to-eat food is now available in many developed countries almost anytime, anywhere. One can buy food not only in restaurants, grocery stores, and coffee bars, but also in gas stations, pharmacies, kiosks, places of work, schools, and in the hospital. We can also have food delivered almost immediately at home or elsewhere. Food which used to be bought in small family-owned stores is now bought in small or large outlets belonging to multi-national corporations with strong marketing skills and vast resources. Improvements in the marketing and distribution of food, as well as food policies such as subsidies of calorie-dense sugar and starch, explain why the total supply of calories has increased tremendously since the 1970s, reaching 3,900 kcal per person and per day in the United States and between 3,400 and 3,600 kcal in other wealthy countries; the exception to this pattern is Japan, where food supply is only 2,700 kcal and where, not coincidentally, obesity is almost nonexistent.^4^

It is true that the metabolism of obese people requires a higher calorie intake and hence that the increased supply of food is a consequence, and not just a cause, of rising obesity rates.^206^ In addition, an increased part of the larger food supply is lost to waste and spoilage, although the estimates of how much is wasted vary between 25% and 40% of the food supply.^207,208^ Still, the increased calorie supply cannot be attributed entirely to increasing food waste or to the higher energy requirements of heavier bodies. In fact, many prominent obesity researchers argue^4^ that the rise in food energy supply is more than sufficient to explain the rise in obesity in the United States from the 1970s.

Access to food is greatly facilitated by the increased availability of ready-to-eat food prepared away from home, particularly in quick-service restaurants. Whereas spending on at-home food remained stable between 1982 and 2007, expenditure on away-from-home food in the United States increased by 16%, and now represents 49% of all food expenditures.^209^ Econometric studies have suggested that the increased availability of fast food (but not full-service restaurants) is a strong predictor of local obesity trends.^18,210,211^ Other studies show that proximity to grocery stores (but not to convenience stores) was associated with a lower BMI, possibly because grocery stores offer more healthful foods.^212^ However, these findings were mitigated by a recent study^213^ which showed that only the proximity to fast-food restaurant significantly influences BMI (particularly for women), whereas proximity to grocery stores or other restaurants does not seem to matter.

**Salience.** In today's cluttered stores and pantries, marketers know that availability, awareness, and even preferences are not sufficient to generate sales; food visibility must be maximized at the point of purchase and at the point of consumption. For example, eye-tracking studies^47,48^ showed that simply increasing the number of facings on a supermarket shelf or placing familiar foods on top of the shelf (versus the bottom) increased the chances that these brands would be noticed, considered, and chosen. One study^214^ found that making healthy foods easier to order at a fast-food restaurant by displaying them conspicuously on the menu led to a significant increase in sales. Displaying healthier food more conspicuously in cafeterias of school lunchrooms (by placing them on eye-level shelves and conveniently at various points in the cafeteria line) also increases their consumption.^86^ Finally, another study conducted at a fast-food restaurant found that a stronger manipulation of salience, asking consumers whether they would like to downsize their side dishes, was accepted by one-third of consumers and was significantly more effective than calorie labeling.^215^ Importantly, the smaller side dishes were not compensated by larger entrees.

The salience (or visibility) of food at home also increases energy intake. When jars of 30 chocolate candies were placed on the desks of secretaries, those in clear jars were consumed 46% more quickly than those in opaque jars.^216^ Another study^32^ showed that simply placing a food magnet on the refrigerator reminding people of food that they had bought in large quantities was enough to trigger consumption of ready-to-eat food. Spreading products in the pantry (versus stacking them) can increase people's awareness that the product is available and increase the likelihood of consumption.^187^ The increased intake of visible foods occurs because their salience serves as a continuously tempting consumption reminder. While part of this may be cognitively based, part is also motivational. Simply seeing or smelling a food can increase reported hunger, devalue other goals, and stimulate salivation and consumption, even when sated.^147,217–219^ Salience can also be generated by asking people to write a detailed description of the last time they ate soup or by asking them when they intend to eat.^220–222^

**Convenience.** One of the strongest trends in food marketing is the focus on improving the convenience of food preparation and consumption. For most people, with the exception of specific festive occasions, food preparation is a cost of inconvenience that consumers are increasingly less willing to pay.^223^ Food marketers have responded to the preference for improved convenience by reducing preparation time and increasing the share of ready-to-eat food. Supporting the role of convenience, studies have shown that increased consumption is largely driven by increased consumption frequency rather than by increased consumption quantity per meal.^223^ The same study showed that between 1978 and 1996 energy intake increased more for snacks (+101%) than for breakfast (+16%), lunch (+21%), and dinner (−37%). The gains were highest among married women who now spend less time preparing food at home. This may also explain why maternal employment is associated with childhood obesity.^224^ Convenience also explains the success of “combo” meals at fast-food restaurants, which combine a sandwich, a side, and a beverage. In fact, one study^225^ showed that consumers place a higher value on a “bundled” combo meal, even after controlling for the effect of price discounts, because they reduce transaction costs and increase the saliency of the “featured” items on the menu board.

Convenience also interacts with other factors such as serving size and salience. In one study,^32^ we stockpiled people's pantries with either large or moderate quantities of eight different foods. We found that stockpiling increased consumption frequency but only for ready-to-eat products, and that this effect leveled off after the eighth day, even though plenty of food remained in stock. Interestingly, we found that stockpiling increased the quantity consumed per consumption occasion of both ready-to-eat and non-ready-to-eat foods throughout the entire two-week period. With ready-to-eat foods, this was due to the higher visibility because of stockpiling.

### Shape and size of serving containers

About 70% of a person's caloric intake is consumed using serving aids such as bowls, plates, glasses, or utensils.^226^ The size of bowls and plates obviously influences energy intake for the 54% of Americans who say that they “clean their plates” no matter how much food they find there.^227^ This can influence energy intake simply because people (and not just those who clean their plates) rely on visual cues to terminate consumption. If a person decides to eat half a bowl of cereal, the size of the bowl will act as a perceptual cue that may influence how much is served and subsequently consumed. Unfortunately, many of these cues are misleading. A number of studies have shown that people in Western societies overestimate the height of a cylindrical object (such as a drinking glass) compared to its width.^228–230^ For example, one of these studies found that the elongation caused people to unknowingly pour and drink 88% more juice or soft drink into a short, wide glass than into a tall, narrow one of the same volume.^229^

Another visual bias, the size-contrast or Delboeuf illusion, suggests that a given amount of product looks smaller on a larger plate than on a smaller plate.^231–233^ A study showed that people who were given 24 oz. bowls of ice cream served and consumed about 20% more ice cream than those given 16 oz. bowls.^234^ Larger serving containers increase consumption even when a constant amount of food is served on the bowl (versus people serving themselves).^30,163^ On the other hand, other studies^235,236^ found that using a smaller plate did not reduce energy intake in lab studies in which subjects were repeatedly eating the identical food in isolation.

Recent studies have started to link these results with work in psychophysics and to look at the interaction effects of size and shape on size perceptions and preferences.^237,238^ An important finding has been that the lack of sensitivity to increasing sizes is even stronger when packages and servings increase in all three dimensions (height, width, and length) compared to when they only increase in one dimension.^191^ This could explain why the effect is stronger for cups, glasses, and bowls (3D objects) than for plates (essentially 2D). The same authors have shown that because people underestimate volume changes that occur in three dimensions, they pour more beverage into conical containers (e.g., cocktail glasses where volume changes in three dimensions) than into cylindrical containers (where volume changes in one dimension). In addition, people's preference for supersizing is higher when products grow in one dimension. Although some studies have shown that part of these effects is mediated by attention,^180,197^ other studies^190,239^ suggest that they are mostly caused by people failing to compound the changes of multiple dimensions.

### Atmospherics of the purchase and consumption environments

Retailers, restaurants, and food service companies can influence the ambient characteristics of the point of purchase and of the point of consumption (e.g., its temperature, lighting, odor, noise, and so on). Some atmospheric dimensions, such as temperature, have direct physiological effects. Studies have shown that people consume more energy when the ambient temperature is outside the thermo neutral zone, the range in which energy expenditure is not required for homeothermy.^240^ For this reason, it has been argued that obesity could be linked to the reduction in the variability in ambient temperature brought about by air conditioning.^241^ For example, consumption increases more during prolonged cold temperatures than in hot temperatures because of the body's need to regulate its core temperature.^242^

Dimmed or soft lighting appears to influence consumption by lengthening eating duration and by increasing comfort and disinhibition. Harsh lighting makes people eat faster and reduces the time they stay in a restaurant, whereas soft or warm lighting (including candlelight) generally causes people to linger and likely enjoy an unplanned dessert or an extra drink.^243,244^ Ambient odors can influence food consumption through taste enhancement or through suppression.^123,245^ For example, one study^147^ found that exposure to an appetizing odor increased soft drink consumption during movie-watching and that exposure to an offensive odor decreased consumption without people being aware of these effects.

The presence of background music is associated with higher food intake^246^ and it is even linked with choice in supermarkets. In the context of restaurants, soft music generally encourages a slower rate of eating, longer meal duration, and higher consumption of both food and drinks.^247^ When appealing music is played, individuals dine longer, feel more comfortable and disinhibited, and are more likely to order a dessert or another drink.^248^ This is because when it improves affective responses (environmental affect, mood or arousal), background music reduces perception of time duration.^249^ In contrast, when music or ambient noise is loud, fast, or discomforting, people tend to spend less time in a restaurant.^250^ A recent meta-analysis found that music also influences shopping in a large range of retail contexts, that slower tempo, lower volume, and familiar music increase shopping duration, whereas loud, fast, disliked music increases perceived time duration.^251^

All of these findings highlight the role of distraction in influencing consumption or intake volume.^58^ For example, one study found that eating while watching TV or eating with friends (but not with strangers) impaired the ability to self-monitor, decreased the attention given to the food itself, and led to higher energy intake.^59^ Other studies found that eating while distracted reduced satiation and impaired memory of past consumption, which reduced the time until the next eating episode.^252^ Indeed, amnesiac patients have been found to eat the same meal multiple times in a row if they are told that it is dinner time.^253,254^ Distraction influences taste perception (e.g., reduces sensory-specific satiety) and increases subsequent consumption volume by emphasizing the affective (versus cognitive) drivers of taste. One study^255^ found that distraction while sampling food increased enjoyment as well as the subsequent choice of the relative vice (chocolate cake) versus the relative virtue (fruit salad).

Although one of the least studied ways marketers can influence consumption, the impact of the eating environment is powerful and multifaceted – and often overlooked by consumers.^201,256^ Overall, these studies show that consumption volume is influenced by the eating environment, by facilitating access to the food, increasing its salience and the convenience of its preparation, but also by modifying the shape and size of serving containers as well as temperature, brightness, ambient odors, and music.

## CONCLUSION

The food manufacturing and retailing industries have evolved tremendously and now include numerous innovative and fast-growing organization that are either nonprofit or with strong concerns for public health and the environment.^257^ However, the majority of the food eaten in developed countries is still manufactured and distributed by traditional for-profit, and often publicly listed, companies.^258^ For-profit food marketers are not focused on making people fat but on making money. In a free market, for-profit food companies that are less profitable than their competitors are likely to end up being acquired by their rivals or to go bankrupt. In this context, the mission assigned to most food marketers is to understand what different consumer segments desire and to profitably offer it to them. In general, what many people want in the short term is tasty, inexpensive, varied, convenient, and healthy foods – roughly in that order of benefit importance. The marketer's mandate is to help identify and create foods that deliver these benefits better; to communicate these benefits; to profitably package, price, and distribute these foods; and to protect these innovations by branding the food so that it acquires unique and positive associations in the mind of consumers. In this respect, food marketers have been very successful and have pioneered many marketing innovations now used in other industries.

Yet, as this review has shown, the vast ingenuity and resources of food marketers have created a myriad of ways in which food marketing influences consumption volume and, hence, may promote obesity. Although television advertising has attracted the bulk of the attention of researchers, it is merely the tip of the iceberg. It is neither the most innovative nor the most powerful way food marketing works, and its importance is declining.

To summarize how food marketing has made us fat, it is most likely through increased access to continuously cheaper, bigger, and tastier calorie-dense food. Two contentions are also offered here: 1) Researchers have overestimated the impact that deliberate decision-making has on food intake. For this reason, the effects of nutrition information, health claims, and informational advertising, have had a smaller impact than is believed. However, this probably does not apply to price and access to food, which are two important influencers of food intake that mostly operate through deliberate decision-making. 2) Researchers have underestimated the impact that peripheral factors and mindless habitual behavior have on food intake. For this reason, the effects of brand associations; calorie density and sensory complexity of food; the size and shape of portions, packages, and serving containers; and the convenience and salience of food stimuli in the eating environment. That is, the effects of the product and the place (the eating environment) have had a greater impact than believed.

### Future research opportunities

Despite decades of work, what we presently know about how food marketing influences consumption is still dwarfed by what we do not know, creating many opportunities for impactful research and ensuring that no review will ever be complete and final. Yet, we should have realistic expectations regarding what research can do. This review shows that food marketing can influence consumption in many inter-related ways and that food consumption is governed by a complex set of dynamic interactions. In this context it is unlikely that any amount of research will be able to “prove” general statements such as “front-of-package health claims improve consumption decisions” because the magnitude and direction of the effects will depend on the implementation and will vary dynamically across consumer segments, consumption occasions, and the type of food studied.

One of the most important areas for future research, therefore, is to examine how the short-term effects reviewed here, which are often investigated only in single-consumption occasions in a lab, also hold when examined across time. Longer time horizons are particularly important because habituation and compensation can offset short-term effects. Ideally, these new studies would combine the best aspects of studies from 1) consumer research (including rich psychological insights and multi-method testing), 2) nutrition (including longitudinal designs, representative participants, biomarkers of calorie intake, and expenditures), and 3) health economics (including population-level interventions and analyses, and policy implications). As such, they would provide the necessary link between specific marketing actions, individual short-term food choices, and long-term population weight gain.

As shown in the tables, the factors leading people to eat more can also lead them to eat less, to promote consumption of healthier food, and more generally increase the importance people attach to health over taste, price, and convenience when making food decisions. For example, we have reviewed studies showing that consumption of healthy and unhealthy food responds similarly to price reductions,^22^ that it is possible to incentivize children to prefer healthier food,^24^ and that smart downsizing can lead people to prefer smaller servings.^191^ In general, there is a wide range of profitable changes that businesses could make to help consumers eat better and eat less. What is important to understand is that these solutions need to fit both supply and demand in the food marketing value chain. In this respect, Tables 1–4 show that much of the leading thinking in this area of win-win approaches has been in food retailing, such as with supermarkets, cafeterias, and restaurants. Thanks to the longer time that consumers spend with food retailers, changes to their marketing have the highest potential to impact consumption.

Finally, it will be important to examine the interplay of marketing factors and cultural, social, and individual characteristics. Although obesity is a global problem, most of the studies reviewed here were conducted among North American consumers and often among undergraduate students. Yet, we know that culture, age, income, education, and a host of other socioeconomic factors influence food decisions. For example, there are important differences between how Americans, Europeans, and Asians approach food and eating. Beliefs that are taken for granted in a US context, for example, that unhealthy food is tastier or that external cues influence satiation, may not apply elsewhere.^113,114,136,259^

### Policy implications

After reviewing the studies outlined here, one may question the effectiveness of the policy changes being suggested by regulators. It is beyond the scope of this paper to examine all the policy interventions designed to fight obesity, and we need to be mindful of the many factors mentioned in the introduction that influence food decisions that are not under the control of food marketers. What this review underscores is that many such changes will come with either modest results or unanticipated results due to how consumers and companies respond. Consider mandatory nutrition information. As a rule, mandatory information disclosure has the intended effect when there is a consensus among consumers about the valence of the information. This occurs when an attribute (like trans-fats, or fibers) is universally seen as negative or positive. However, mandatory disclosure may backfire if the information is about attributes that are not uniformly valued – like calories, salt, fat, or sugar content – which are seen by some as a signal of rich taste. In this case, companies may actually choose to compete on less transparent attributes like taste and to target taste-conscious consumers.^132^

By highlighting the effects of unobtrusive environmental factors on energy intake, the findings in this review support the current “small steps” approach to obesity prevention.^260^ This approach recognizes that obesity is not a moral weakness but a normal response to the changing environment. As such, it stands in contrast with traditional public health efforts that have focused on providing science-based nutrition information and have exhorted people through didactic and sometimes moralizing appeals to change their dietary habits. A small steps approach focuses on adopting smaller, more sustainable goals. It recognizes that self-control is a limited and often absent resource and focuses less on persuasion and more on benevolent interventions that “nudge” consumers into making slightly better but repeated food choices without thinking about it.^261^ This is done mostly by altering the eating environment, for example, by substituting calorie-dense drinks, like soft drinks, with water or diet soft drink in cafeterias, surreptitiously improving food composition, indirectly promoting smaller packages on menus (by eliminating quantity discounts and adding an extra small size to the range), storing tempting food out of reach and healthier alternatives within reach, using smaller cups and bowls, and pre-plating food instead of using family-style service. The small steps approach is not designed to achieve major weight loss among the obese but to prevent obesity for the 90% of the population that is gradually becoming fat by eating 60–100 calories too many per day.^262,263^ It should be paired with smarter public education campaigns to rebrand health by associating it with stronger identity-based appeals, such as sustainability, animal welfare, or even national security.^264^
